# Polychlorinated Alkane Profiles and Concentrations in Bolivian Andes Soils Point to a Long-Range Transport Influence

**DOI:** 10.1021/acs.est.5c14672

**Published:** 2026-03-11

**Authors:** Bo Yuan, Cheng Wu, Cynthia A. de Wit, Claudia Mohr, Marcos Andrade, Isabel Moreno, Volker Brüchert, Rienk Smittenberg, Matthew MacLeod

**Affiliations:** † Department of Environmental Science (ACES), Stockholm University, Stockholm 106 91, Sweden; ‡ Department of Chemistry and Biomedical Science, Norwegian University of Science and Technology (NTNU), Trondheim 7491, Norway; § Department of Chemistry and Molecular Biology, Atmospheric Science, 3570 University of Gothenburg, Gothenburg 413 90, Sweden; ∥ Department of Environmental Systems Science, ETH Zürich, Zürich 8092, Switzerland; ⊥ PSI Center for Energy and Environmental Research, Paul Scherrer Institute, Villigen 5232, Switzerland; # Laboratory for Atmospheric Physics, Institute for Physics Research, 28147 Universidad Mayor de San Andrés, La Paz 4787, Bolivia; 7 Department of Atmospheric and Oceanic Sciences, University of Maryland, College Park, Maryland 20742, United States; 8 Instituto de Investigaciones Fármaco Bioquímicas, 28147 Universidad Mayor de San Andrés, Av. Saavedra N 2224, La Paz 4787, Bolivia; 9 Department of Geological Sciences, Stockholm University, Stockholm 106 91, Sweden; 10 Bolin Centre for Climate Research, Stockholm University, Stockholm 106 91, Sweden; 11 28500 Swiss Federal Institute for Forest, Snow and Landscape Research, Birmensdorf 8903, Switzerland

**Keywords:** High-altitude environment, urban transport, altitudinal trend, chlorinated paraffins, long-range transport

## Abstract

High-altitude terrain may intersect the upper atmospheric boundary layer and exhibit distinct environmental dynamics. We investigated the anthropogenic pollutants polychlorinated alkanes (PCAs, also known as chlorinated paraffins) in surface soils along a transect from the La Paz-El Alto metropolitan area in Bolivia (3200–4100 masl) to the upper slopes of Mount Chacaltaya (>5200 masl), around 16 km away. Concentrations of PCAs in urban soils (750–5,230 ng/g organic carbon [OC]) decreased exponentially with increasing distance from the urban boundary, declining to ∼150 ng/g OC at elevations below 4,700 masl. Beyond 4,700 masl concentrations increased again, reaching levels comparable to those in the urban area, 1,670–4,300 ng/g OC, above 5,000 masl. Given that pollutant concentrations typically decline with distance from their source, this altitudinal trend, together with a pronounced shift in PCA forensic fingerprints near 4,700 masl, strongly suggests contributions from sources beyond the local metropolitan area. Carbon and nitrogen isotope signatures in organic carbon further support long-range transport as a source, consistent with previous modeling and observations that the upper slopes of Mount Chacaltaya predominantly receive air masses and organic carbon from distant regions via transport in the free troposphere. Our observation that pollutant levels in high-altitude areas are comparable to those in the metropolis of 1.8-million inhabitants underscores the efficiency of long-range atmospheric transport.

## Graphical abstract

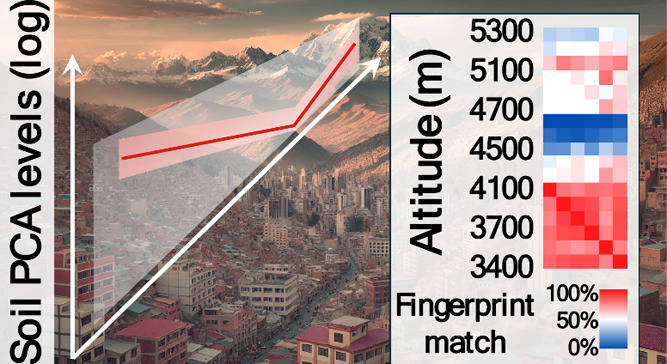

## Introduction

High mountains cover 24.3% of the global land area and provide essential ecosystem services, including freshwater resources for hundreds of millions of people.^1,2^ For example, cities like Denver and Mexico City depend on snowmelt from mountains >3,000 m above sea level (masl),^3^ and in Bolivia more than 25% of the population resides above 3,500 m.^4^ These regions are characterized by hypobaric hypoxia, cold and dry conditions, and intense solar radiation, which make high-altitude ecosystems particularly vulnerable to anthropogenic stressors.^4^

Although often considered remote and pristine, mountain environments are increasingly recognized as sinks for anthropogenic pollutants. Semivolatile organic chemicals can undergo long-range atmospheric transport and accumulate in high-altitude ecosystems through cold trapping, with soils acting as important reservoirs due to their high organic carbon content.^5,6^ Elevated concentrations of persistent organic pollutants (POPs) such as endosulfan and hexachlorocyclohexanes have been reported above 5,000 masl,^7^ and even higher atmospheric levels of emerging contaminants, including brominated flame retardants, organophosphate esters, and perfluoroalkyl substances, have been observed at >5,000 masl compared to nearby urban areas.^8^ Such findings highlight the susceptibility of mountain ecosystems to contamination from distant sources, with implications for both biodiversity and human exposure in high-altitude regions.^9,10^

A unique opportunity to study persistent organic pollutants at high altitudes is presented by the metropolis-to-mountain setting in La Paz, Bolivia. The La Paz and El Alto metropolitan area is home to approximately 1.8 million people, and is located at elevations between 3200 and 4100 masl.^11^ Just 16 km to the north and 1,550 m higher in elevation lies the Chacaltaya Global Atmospheric Watch station (site code: CHC) at 5,240 masl. As a large urban center, La Paz is a significant source of chemical pollutants. In contrast, CHC is classified as a background site within the Global Atmospheric Passive Sampling Network.^8^ This classification is supported by 4-day air mass back trajectory modeling using FLEXPART, which shows that only about 24% of the air sampled at CHC originated within the lowest 1.5 km of the atmosphere, representing the boundary layer.^12^ While this fraction may include contributions from surface-level sources such as the metropolitan area, it does not exclusively indicate direct urban influence.^13–15^ The remaining 76% of air masses arrive at CHC from the free troposphere, generally traveling between 100 km and over 1,000 km before arrival, with only a few cases representing pure free-tropospheric air.^16^

We selected polychlorinated alkanes (PCAs), also known as chlorinated paraffins, as the focus of this study due to their widespread use, environmental persistence, and distinct forensic chemical fingerprints. PCAs are common chemical additives that are incorporated into consumer products,^17^ construction materials,^18^ and polymer-based materials.^19^ They have been identified as accounting for 92% of extractable organochlorine substances in municipal wastewater sludge^20^ and have been detected at concentrations one to 3 orders of magnitude higher than those of other POPs in remote soils worldwide.^19^ PCAs are synthesized from paraffins with varying alkane chain lengths and are classified into four categories: very-short-chain (C_6–9_), short-chain (C_10–13_), medium-chain (C_14–17_), and long-chain (C_>17_). Their general chemical formula, C_*x*_H_2x+2‑y_Cl_*y*_ (*x* = 6–40, *y* ≥ 1), encompasses 600–700 C_*x*_Cl_*y*_ homologues, and distinct homologue fingerprints have been used in environmental forensic analysis to trace contamination origins.^21,22^

Previous high-altitude studies on the Tibetan Plateau examined short-chain^23^ and medium-chain PCAs^24^ in air (1983–4553 m a.s.l.) and reported increasing atmospheric concentrations with elevation, suggesting potential inputs into alpine ecosystems. However, these studies were limited to air samples collected at urban and remote mountain sites without direct urban-mountain connections, leaving a gap in evidence from soils and other alpine compartments that are considered more indicative of pollutant enrichment.^25^

In this study, we extend the elevation range to 3438–5225 m a.s.l. and analyze surface soil samples collected along a transect from La Paz-El Alto to CHC. By comprehensively covering PCAs from very-short- to long-chain groups, we assess spatial trends from an urban source to a high-altitude environment. Using urban signals as benchmarks, we further evaluate the relative contributions of local emissions versus long-range transport to pollutant accumulation. This provides the first systematic evidence of PCAs in South American alpine soils, addressing critical knowledge gaps regarding emerging contaminants in high-altitude ecosystems.

## Methods

### Sample Collection

The sampling campaign was conducted in May 2018, precisely corresponding to the time modeled in FLEXPART simulations^12,15^ for Chacaltaya. Samples were collected from the top 5 cm of surface soils. Eight relatively undisturbed garden soil samples were collected from urban La Paz – El Alto within a 2-km distance, covering an altitude range of 3,400–4,100 masl. These urban sampling sites represent two densely inhabited sectors of La Paz-El Alto, which are separated by steep local topography that limits urban development. The center of the studied urban area was defined based on the geometric mean of the eight urban sampling points, with the urban boundary set at 2 km from the studied urban center and onward. Distances were calculated using GPS coordinates. Additionally, 15 soil samples were collected along an altitudinal transect toward the summit of Chacaltaya (4,200–5,100 masl), with sampling sites selected away from public dirt roads to minimize potential disturbances from vehicle emissions and road dust. Five high-altitude soil samples were collected at approximately 5,200 masl. The altitude along the transect increased linearly with distance from the studied urban center according to the following relationship:

$$\mathrm{Altitude}\;\left(m\right)=100\times \mathrm{Distance}\;\left(\mathrm{km}\right)+3466\;\left(r^{2}>0.98\right)$$ 1

Thus, each kilometer traveled from the urban center corresponds to an altitude increase of approximately 100 m. To assess potential differences in samples unrelated to altitude effects, two additional soil samples were collected along a westward transect of the urban center at ∼4,000 masl. All samples were placed in Whirl-Pak bags and stored at −20 °C until further processing. Detailed GPS coordinates, altitude, and distance from the center of the studied urban area for all soil sampling sites are provided in Table S1, with their locations shown on the site map in Figure S1.

### Sample Preparation

Soil samples were freeze-dried, and water content was determined. After sieving, a subset of samples for total organic carbon (TOC), total organic nitrogen (TON), and stable isotope (δ^13^C and δ^15^N) analyses were further dried at 105 °C for 4 h. For these analyses, 30–50 mg of ground, dried soil was packed into silver capsules and analyzed using an elemental analyzer (Flash-EA 1112, Thermo Fisher Scientific, San Jose, CA) coupled to an isotope ratio mass spectrometer (IRMS, Delta V, Thermo Fisher Scientific, San Jose, CA).^26^

The preparation method for analysis of PCAs was adapted from an established protocol.^27^ Briefly, ∼15 g of dry soil was spiked with 10 ng of ^13^C-1,5,5,6,6,10-hexachlorodecane (Cambridge Isotope Laboratories, Andover, MA, USA) as an internal standard and extracted using an Accelerated Solvent Extractor (ASE 300; Dionex Europe, Leeds, UK). Activated copper was added to remove sulfur. The extracts were then purified using a multilayer solid phase extraction (SPE) column. Dechlorane 603 (20 ng) was used as an injection standard and was added prior to instrumental analysis.

### Pollutant Analysis

PCAs were analyzed based on C_*x*_Cl_*y*_ homologues using direct injection dichloromethane (DCM)-enhanced APCI-Orbitrap HRMS (Q Exactive, Thermo Fisher Scientific, San Jose, CA).^28^ The instrument operated in full-scan mode (*m*/*z* 250–2000) with a resolution of 120,000 (full width at half-maximum). The injection volume was 3 μL, with a mobile phase flow rate of 0.100 mL/min and a DCM flow rate of 0.010 mL/min. Additional parameters included a maximum ion injection time of 250 ms, an automatic gain control target of 5e10^6^, a sheath gas flow rate of 17 arbitrary units (arb), and an auxiliary gas flow rate of 1 arb. To maximize detection of volatile homologues, the following settings were applied: capillary temperature at 150 °C, auxiliary gas heater temperature at 150 °C, and a spray current of 4.5 μA. Over 400 C_*x*_Cl_*y*_ homologues (ranging from C_6_Cl_4_ to C_48_Cl_12_) were screened to construct the C_*x*_Cl_*y*_ fingerprint profile. Quantification of very-short-chain (C_<10_), short-chain (C_10–13_), medium-chain (C_14–17_), and long-chain PCAs (C_>17_) was performed using C_*x*_Cl_*y*_ profile reconstruction using 1, 5, 7, and 5 reference standards, respectively,^22^ as detailed in Table S2 The analysis followed the reporting format recommended by Fernandes et al.^29^

### Quality Assurance and Quality Control

The recovery of the internal standard was 75 ± 20%. PCA homologue profiles were satisfactorily reconstructed across the samples with the goodness-of-fit R^2^ ranging between 0.50 and 0.98. Two field blanks were included to assess potential field and transport contamination, each using 15 precleaned polyurethane foam plugs. The field blanks were analyzed alongside the samples. Sample preparation was conducted inside a laminar flow clean hood to maintain contamination-free conditions. Each batch of samples for preparation also included a procedural blank. The method detection limit (MDL) for each PCA class was calculated as the mean procedural blank plus three times the standard deviation. The MDLs per sample were 0.42 ng for PCAs-C_6–9_, 5.0 ng for PCAs-C_10–13_, 7.8 ng for PCAs-C_14–17_, and 0.19 ng for PCAs-C_>17_, respectively, varying depending on the weight of the individual analyzed sample. Duplicate tests were performed for δ^13^C in nine soil samples, with a mean percent difference of 0.89% of 1‰.

### Forensic Fingerprinting

The goodness-of-fit R^2^, used to evaluate reconstruction performance in quantification, reflects the similarity between the C_*x*_Cl_*y*_ fingerprints of two compared samples.^21^ In this study, the approach was applied for pairwise comparison of soil fingerprints. R^2^ values range from 0 to 100%, where R^2^ = 100% indicates identical fingerprint profiles.

### Statistical Analysis

Linear and nonlinear fitting, correlation and statistical analysis were performed using PAST Version 5.0.^30^ A significance threshold of *p* = 0.05 was applied. Colormap contours of PCA concentrations were generated using OriginPro 8.5. For pollutant concentrations below the MDL, values were assigned as MDL/√2.

## Results

Results for the individual soil samples are given in Table S1 in the Supporting Information. This includes stable isotope ratios (δ^13^C, δ^15^N), OC and organic nitrogen (ON) contents, dry weight concentrations, and OC-normalized concentrations. While dry weight concentrations are reported in Text S1 and Figures S2–S3 for reference, OC-normalized concentrations were used throughout the main analysis as they provide a more robust basis for assessing altitudinal trends.

### Soil Organic Matter Characterization

TOC decreased with increasing distance and altitude, from 2.82 ± 2.54% in urban soils to 0.31 ± 0.13% in soils above 5,000 masl, reflecting the sandy soil structure and eroded rocky terrain near the summit. The decrease in TON was less pronounced, with a mean value of 0.27%, fluctuating between 0.10% and 0.90%. Both δ^13^C and δ^15^N exhibited a decreasing trend, indicating an increasing abundance of lighter isotopes closer to the summit (mean δ^13^C: −24.68‰, δ^15^N: + 5.51‰). Significant correlations were found between TOC and dry weight-based PCA concentrations (*p* < 0.01), supporting the use of TOC normalization to examine altitudinal trends.^31^

### Urban-to-Mountain PCA Trends

OC-normalized concentrations plotted against GPS coordinates are shown in Figure 1, with altitude indicated by contour lines. The figure illustrates the declining concentrations with distance from the urban area followed by increases with altitude, highlighting PCA enrichment toward the summit. The urban area is represented by two spatial hotspots (Figure 1), which correspond to distinct sectors of La Paz–El Alto separated by intervening steep terrain that constrains urban development (Figure S1). On average, OC-normalized sumPCA concentrations were 2,240 ng/g OC in urban soils, 309 ng/g OC along the mountain transect, and 2,440 ng/g OC above 5,000 masl. Using urban soils as benchmarks, PCAs-C_6–9_ and PCAs-C_10–13_ decreased to 42% and 27% of mean urban concentrations along the transect but exhibited a 344% and 187% increase, respectively, in high-altitude soils, reaching means of 111 and 1,070 ng/g OC. PCAs-C_14–17_, which declined to only 10% of urban levels along the trail, increased to a comparable concentration at high altitudes (1,200 ng/g OC) relative to urban soils (1,390 ng/g OC). Following this pattern, the PCAs-C_>17_ declined even more sharply, dropping to <1% of urban levels along the trail but still reaching 25% of mean urban concentrations (240 ng/g OC) above 5000 masl (59 ng/g OC). The turning point in concentrations was observed at approximately 4,700 masl (Figure S4). Below and above this altitude, log-transformed, OC-normalized PCA concentrations exhibited statistically significant decreasing and increasing trends with altitude, respectively (*p* < 0.01 in 7 out of 8 regressions, and <0.05 in the other case; Figure 2).

**Figure 1:**
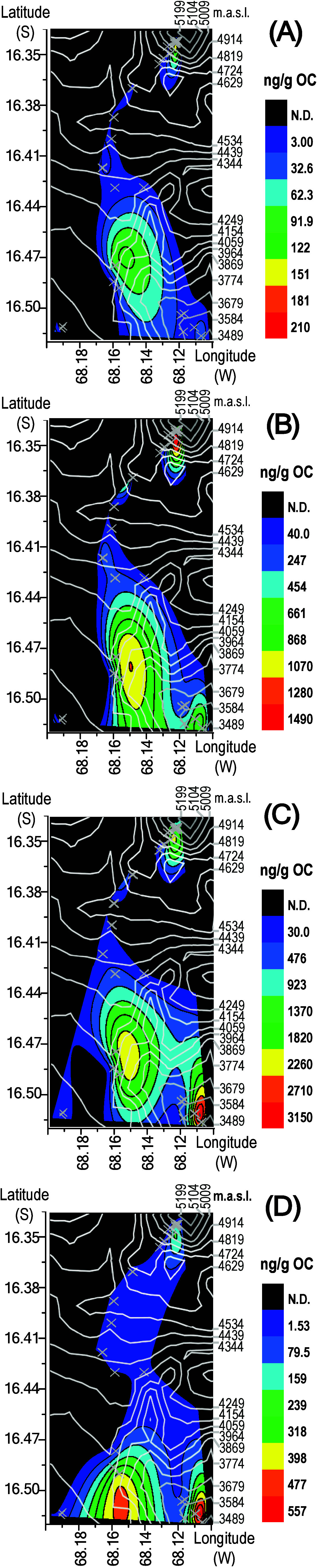
Spatial distribution of OC-normalized soil concentrations for (A) PCAs-C_6–9_, (B) PCAs-C_10–13_, (C) PCAs-C_14–17_, and (D) PCAs-C_>17_ plotted against GPS coordinates. Altitude is indicated by contour lines. Crosses mark soil sampling locations, while black areas are labeled ″N.D.″ indicating regions with no data (i.e., no samples collected). The two urban hot spots at the bottom represent distinct sectors of La Paz-El Alto separated by steep local topography that constrains urban development, and the hot spot at the top corresponds to the Chacaltaya summit.

**Figure 2:**
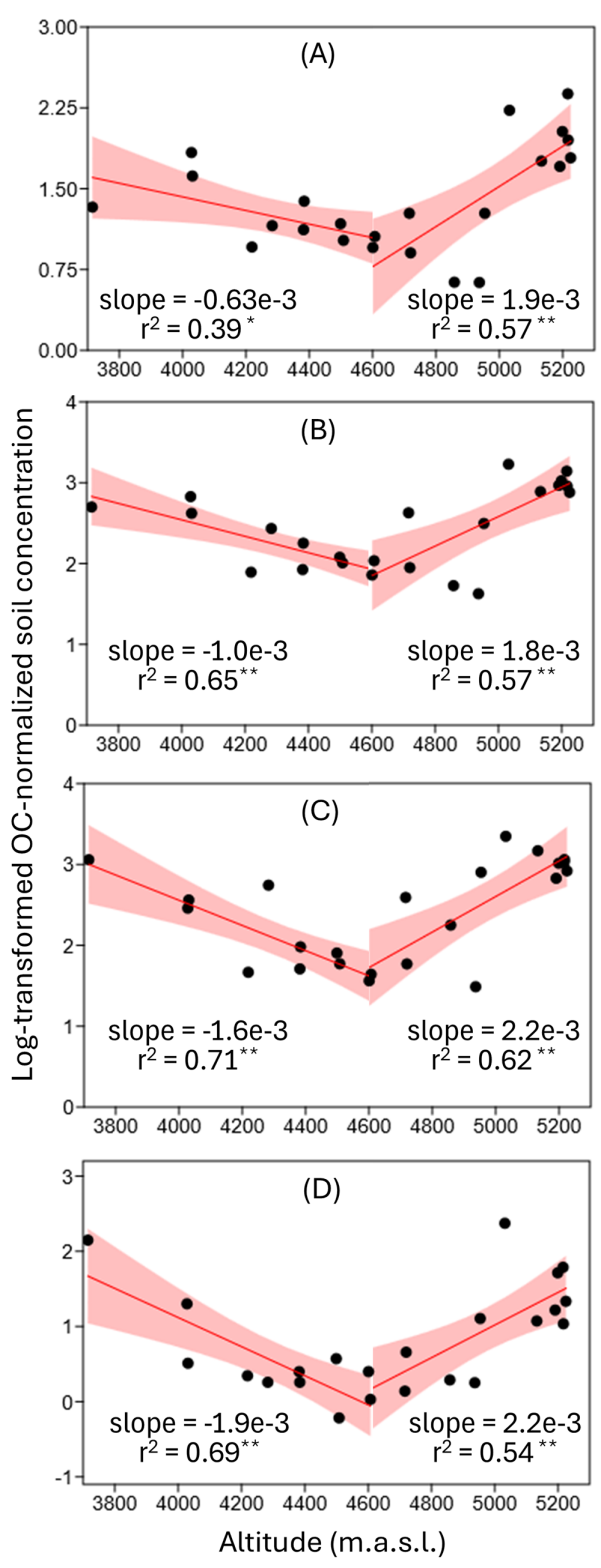
Altitudinal trends of log-transformed OC-normalized (A) PCAs-C_6–9_, (B) PCAs-C_10–13_, (C) PCAs-C_14–17_, and (D) PCAs-C_>17_ concentrations (ng/g OC) modeled using two linear correlations with a breakpoint below 4700 masl identified from the data analysis. Urban sample concentrations and altitudes are represented as geometric means, approximating the urban area as a single source point for fitting purposes. *p* < 0.01 (**), *p* < 0.05 (*). For fitted regression equations see Equations S5–S12.

### Forensic Fingerprinting

A total of 191 C_*x*_Cl_*y*_ homologues were identified across the soil samples, with carbon chain lengths ranging from C_6_ to C_25_. Pairwise correlation matrix analysis, using the 191 C_*x*_Cl_*y*_ homologue abundances as fingerprints for individual samples (Figure 3), revealed fingerprint patterns that grouped the samples into three categories: urban soils, transect soils between 4,000 and 5,100 masl, and summit soils around 5,200 masl. Pairwise comparisons of PCA homologue fingerprints along the altitude gradient showed a gradual shift in similarity between neighboring altitude sites, with R^2^ values above 60% (as illustrated in the similarity analysis in Figure 4). However, a sharp transition occurred around 4700 and 4,800 masl, where similarity dropped below 40% (29–36%), before stabilizing around 60% again up to the summit.

**Figure 3:**
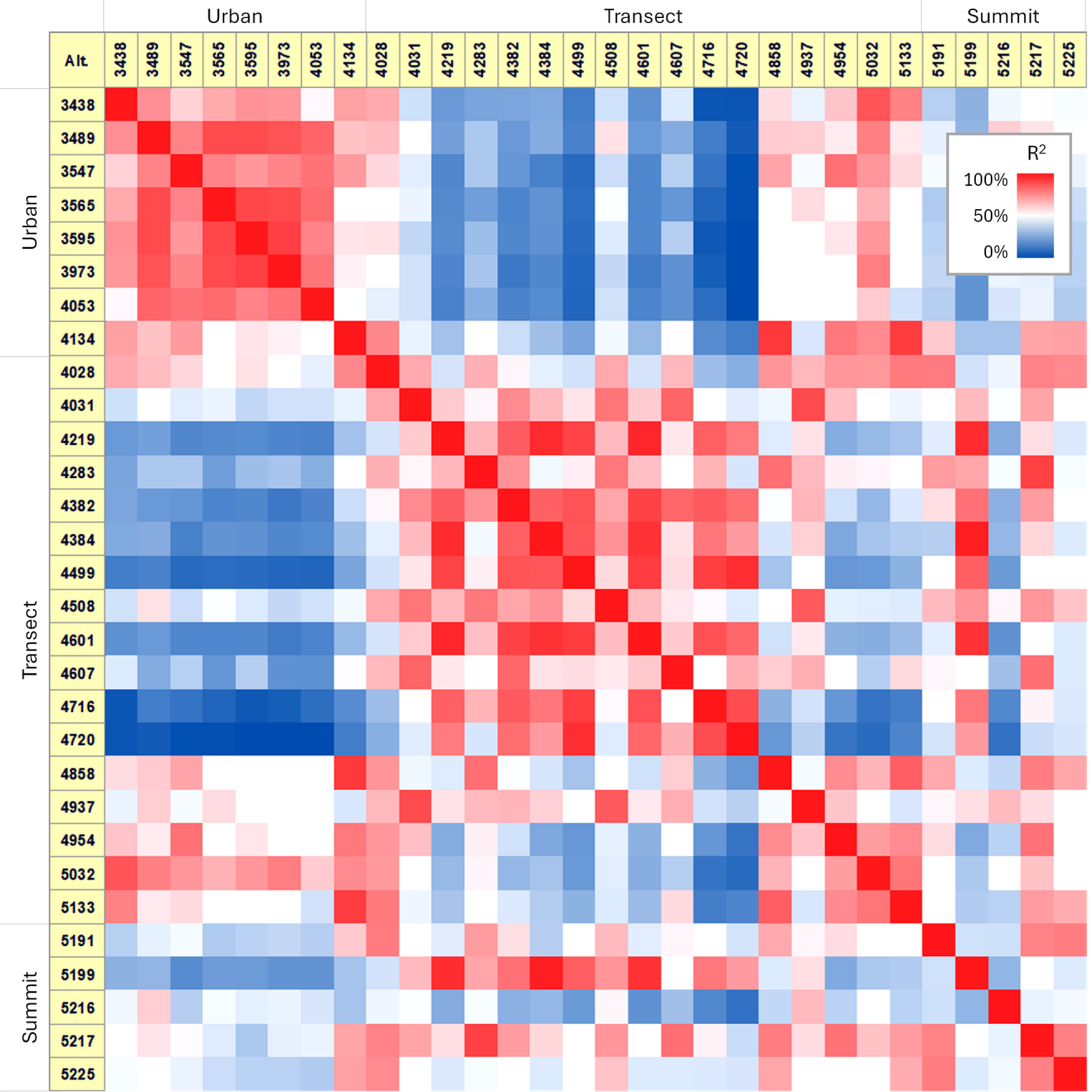
Pairwise forensic comparisons of PCA fingerprints along altitude. R^2^ indicates how closely the PCA fingerprints resemble one another. Larger R^2^ values imply consistent source signatures or comparable fractionation histories, whereas lower values indicate apparent shifts in fingerprints, such as changes in sources with altitude.

**Figure 4:**
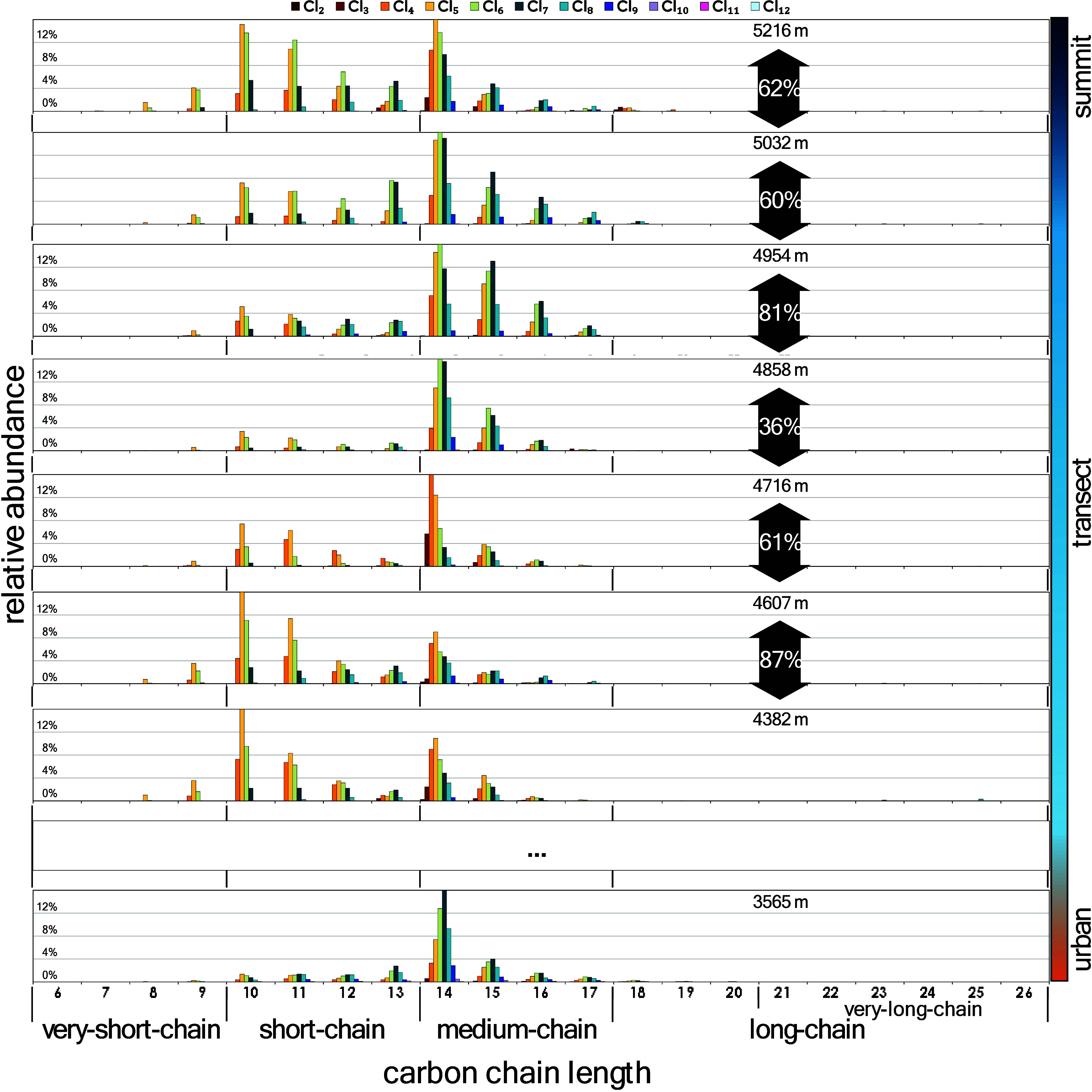
Development of PCA fingerprints in soil samples collected across different altitudes, showing both forensic similarity comparisons between neighboring altitude intervals (R^2^ values) and the relative distributions of individual homologues.

As shown by the homologue fingerprints in Figure 4, up to ∼4,700 masl, the fingerprints were dominated by relatively volatile homologues, with increasing relative abundances observed for species such as C_9_Cl_4–5_, C_10_Cl_4–5_, C_11_Cl_4–5_, C_12_Cl_4_, and C_14_Cl_3–4_ within their respective carbon groups. Above this altitude, higher-chlorinated homologues became increasingly dominant, particularly C_9_Cl_5–6_, C_10–12_Cl_5–6_, C_13_Cl_6–7_, and C_14–17_Cl_5–6_, surpassing the less chlorinated forms within each carbon group. In addition, long-chain homologues (C_18–20_) reappeared and became more abundant at ∼5,000 masl and above.

When plotting the relative abundances of C_10_-C_16_, which were consistently detected across samples, the enrichment pattern, reflected by the slope (Table S4), did not always follow a monotonic decrease with increasing carbon chain length or decreasing volatility. Notably, C_14_ exhibited the most distinct enrichment trend, showing the steepest slope among all carbon groups.

## Discussion

### Urban Source of Anthropogenic Pollutants

Urban areas are important sources of anthropogenic chemical pollutants, as evidenced by the significant correlation between PCA concentrations in surface soils^32^ and ambient air^33^ with local population density in previous studies. In our study, PCA levels in urban soils were significantly higher than those in transect soils and formed distinct hotspots within the Bolivian highland area. This finding reinforces that urban La Paz-El Alto, home to 1.8-million people, is a clear local source of these chemicals in the Andes.

PCA concentrations in urban soils and soils near known sources have predominantly been reported on a dry-weight basis in the existing literature. Therefore, our measurements from soils collected in and around the city of La Paz – El Alto are also presented on a dry-weight basis to facilitate direct comparison. Median (range) concentrations for PCA-C_10–13_ and PCA-C_14–17_ were 14.8 (4.25–34.8) ng/g d.w. and 29.7 (9.17–81.3) ng/g d.w., respectively, comparable to values reported in global urban soils, although maximum concentrations in La Paz–El Alto were lower than those observed in other urban areas. In Shanghai, China (2011), urban soil concentrations ranged from ND–615 ng/g d.w. for PCAs-C_10–13_ and 1.95–188 ng/g d.w. for PCAs-C_14–17_.^34^ Similarly, PCA concentrations in Tibetan soils, another highland environment, were within a comparable range: 4.0–188.9 ng/g d.w. for PCAs-C_10–13_ and 2.6–139.3 ng/g d.w. PCAs-C_14–17_. PCAs-C_14–17_ were the dominant group in urban La Paz soils, with concentrations 1.6 – 3.8 times higher than PCAs-C_10–13_, a pattern similar to that observed in European urban areas. In Swiss urban settlements (1988 – 2014), concentrations ranged from 3.0–35 ng/g d.w. for PCAs-C_10–13_ and 5.1–160 ng/g d.w. for PCAs-C_14–17_.^35^ In general, PCA concentrations in soils in the La Paz-El Alto urban area fall within the range of other global urban soils and remain far lower than levels at direct industrial sources such as production sites^36,37^ or e-waste facilities.^38^

There are major knowledge gaps regarding the presence of PCAs-C_6–9_ and PCAs-C_>17_ in soils. In this study, their median (range) concentrations were 0.72 (<MDL–1.85) ng/g d.w. and 8.58 (0.19–15.1) ng/g d.w., respectively. A 2018–2019 study on German forest soils reported PCA-C_6–9_ and PCA-C_>17_ concentrations of 1.5 (0.67–3.9) ng/g d.w. and 9.5 (1.9–33) ng/g d.w., respectively.^39^ PCA-C_10–13_ and PCA-C_14–17_ concentrations in the same study were also in a comparable range, at 27 (7.7–99) ng/g d.w. and 18 (11–49) ng/g d.w., respectively. Unlike the German soil samples, where very-long-chain PCAs were universally present, these compounds were rarely detected in the studied Bolivian soils, with the longest identified PCAs reaching only C_25_.

### Transport Patterns at High Altitude

The observed spatial trends of PCAs-C_6–9_ and PCAs-C_>17_ complemented insights into PCA dispersion at high altitudes gained from PCAs-C_10–13_ and PCAs-C_14–17_. PCAs-C_6–9_, the most volatile PCA group, exhibited only a 14.5% dry weight concentration decline every two kilometers, suggesting a higher potential for regional transport. This finding aligns with a German study, which demonstrated that PCAs-C_6–9_ exhibited the most diffuse spatial distribution compared to other PCA groups.^39^ Spatial distribution patterns observed previously in flat terrain near sea level suggest that PCAs-C_10–13_ and PCAs-C_14–17_ concentrations remain relatively stable within a 4 km radius from their emission source.^36^ A similar but slightly extended pattern was observed in another highland study on the Tibetan Plateau, where PCA-C_10–13_ concentrations remained relatively level beyond 5 km from the source, likely influenced by dynamic airflow along mountain/valley wind directions.^40^ In the present study, dry weight concentrations of PCAs-C_10–13_ exhibited a steady decline of ∼38% every two kilometers beyond the urban sampling center, extending to the farthest sampling site (15 km), while PCA-C_14–17_ stabilized within a 4 km distance from the urban region (Figure S2). This trend may be influenced by altitude-dependent enrichment, as concentrations increased by ∼170% every two kilometers (200 m altitude increase equivalence as per eq 1) away from the urban sampling center. When both trends were considered together, PCA concentrations remained approximately consistent beyond the 4 km from the urban center (Table S5). Similar to PCAs-C_14–17_, PCAs-C_>17_, the least volatile group, stabilized at relatively constant levels beyond the 4 km radius from the urban boundary (Table S1).

In the studied Andean region, transport through the atmospheric boundary layer (ABL) could be a key driver of pollutant dispersion from the urban source, with its daily dynamics and expansion strongly influenced by high solar radiation and the semiarid conditions of the Altiplano.^13^ This results in a largely bidirectional transport pattern throughout the year, primarily due to the daily expansion of the ABL, with upslope winds following the canyon’s orientation and blowing mainly from the south.^41^ However, the overall dispersion range is limited, and upslope winds are not efficient mechanisms for the long-term transfer of PCAs, especially for the least volatile PCAs (C_14–17_ and C_>17_), to high-altitude sites. Consequently, the contamination observed in summit soils is more consistently explained by long-term deposition processes^42^ and remote source inputs, as supported by FLEXPART analyses.^12^

### Cold Trapping and Deviating Fractionation Patterns

Organic pollutant concentration trends and fractionation patterns at high altitudes are often evaluated using the “mountain cold trapping” conceptual model, in which there is enhanced wet deposition at lower temperatures, especially via precipitation.^5^ Mountain cold trapping is most effective for pollutants with log K_WA_ values between 3.5 and 6 and log K_OA_ values between 8.5 and 11.5, and can lead to increasing concentrations with altitude, as observed for PCB-153.^5^ In contrast, less volatile pollutants (log K_OA_ > 12) tend to deposit efficiently at lower altitudes, and more volatile chemicals (log K_OA_ < 8) do not deposit at any temperature, and thus do not follow a cold trapping trend. Based on the physicochemical properties of C_10_ to C_20_ PCA homologues, log K_OA_ and log K_WA_ values range from 4.24 to 21.74 and −1.38 to 8.45, respectively.^43^ Within this range, a subset of medium-chlorinated, short-chain PCAs falls into the partitioning window characteristic of cold trapping. This is supported by the increasing proportion of C_10_Cl_5–7_ and C_11_Cl_5–6_ homologues, observed in fingerprint profiles with altitude (Figure 4). These homologues appear to behave similarly to PCB-153, a pattern also reflected in dust–air partitioning studies.^44^ Such behavior may help explain the weak enrichment patterns previously reported for PCAs at similar altitudes in a global soil study.^6^

The short and comparable urban transport distances of PCAs-C_14–17_ and PCAs-C_>17_ (Figures S2C and S2D) align with cold trapping theory, which predicts that these low volatile pollutants deposit efficiently nearby their urban source. However, despite their log K_OA_ values exceeding the ideal range for cold trapping, both PCAs-C_14–17_ and PCAs-C_>17_ still exhibited significant enrichment at high altitudes when normalized to soil OC-content. This suggests the presence of an additional source of pollutants, with the highest concentrations at high altitudes potentially pointing toward its origin.

Below and above the transition altitude of approximately 4,700 masl, OC-normalized PCA concentrations exhibited significant increases bidirectionally toward both the urban area and the summit (*p* < 0.01 in 7 out of 8 regressions, and <0.05 in the other case; Figure 2). This turning point likely reflects the approximate upper reach of La Paz’s boundary-layer influence, since the city lies from ∼3,200 masl^11^ and, according to FLEXPART simulations,^12^ the pseudoboundary layer extends about 1.5 km above ground level, corresponding to ∼4,700 masl. Above this level, remote contributions progressively dominate. The upward trend at higher altitudes is unlikely to be driven by local sources, as no permanent human activities or settlements exist near the summit and La Paz-El Alto is the only major nearby urban source. Combined with the observed fingerprint deviations from expected fractionation and FLEXPART simulations showing minimal near-field influence, this supports the interpretation that summit contamination cannot be explained by local emissions or cold trapping alone.

Notably, among all carbon chain lengths, PCAs-C_14_ exhibited the highest enrichment (Table S4), while PCAs-C_>17_ reappeared above detection limits as altitude increased (Figure 4). Typically, PCA fractionation along altitudinal gradients is expected to reflect homologue-specific physicochemical properties, primarily linked to carbon chain length and volatility.^45^ However, the observed PCA profiles at high altitudes showed a distinct shift toward a different fingerprint pattern starting from approximately 4,700 masl (Figure 4). This shift, together with the pronounced V-shaped altitudinal trend in PCA concentrations (Figure 2), and supported by FLEXPART modeling, strongly indicates a transition between distinct PCA sources rather than continuous dispersion from the urban area at lower elevations.

### Sources beyond the Nearby Urban Area

Like other anthropogenic pollutants, PCAs have no natural sources. Their persistence enables them to reach remote areas with no apparent local emissions, making them global pollutants. For example, a recent Tibetan Plateau study found PCAs in remote Chinese lake sediments, traced back to emissions from India,^46^ illustrating the potential for long-range atmospheric transport. This mechanism may likewise contribute to the elevated PCA levels and distinct PCA profiles at the high-altitude sites in the present study.

Compared to many other high-altitude stations, the site at CHC benefits from extensive research on air mass origins. At Chacaltaya’s upper slopes, transport from the Altiplano boundary layer occurs frequently, evidenced by weekly pollutant cycles.^47^ Nevertheless, in addition to the FLEXPART modeling results, which indicate that only 24% of air masses at Chacaltaya had recent contact with the nearby surface,^12^ another study found that 71% of the OC at the site originates from distant sources, with only 29% attributable to the nearby metropolis.^14^ For instance, transport from regions 200 km away or more, such as smoke from burning lowlands,^16^ has been documented, in agreement with regional pollen observations showing species typically found within a ∼300 km radius.^48^ Longer range air masses arriving via free-tropospheric (FT) flows from the western marine region^49^ and Amazonia,^50^ both over 1,000 km away, have been recorded at Chacaltaya. Such long-range transport pathways also explain the interhemispheric gaseous mercury exchange observed at the site.^51^

These findings align with the δ^13^C and δ^15^N isotope signatures in the present study (Table S1), which align with aerosol fractionation values previously observed from Santarem Brazil,^52^ suggesting a potential organic matter source. Particulates including microplastics^53^ and black carbon,^54^ have recently been shown capable of traveling long distances in the FT, indicating that PCAs, especially less volatile homologues PCAs-C_14–17_ and PCAs-C_>17_ could also be transported over considerable ranges despite their larger molecular size. Collectively, these observations support the hypothesis that PCAs found at Chacaltaya could originate from sources beyond the immediate metropolis.

Although the current soil data set alone cannot conclusively confirm remote PCA sources at Chacaltaya, largely due to the scarcity of PCA data across the South American continent, it nonetheless provides strong evidence of such influence. When considered together with site-specific FLEXPART modeling,^12^ the case for remote sources dominating becomes compelling. The limited regional PCA fingerprints available offer only preliminary evidence that PCAs-C_14–17_ and PCAs-C_>17_ homologues in this study may be linked to multinational sources. For instance, PCAs-C_14–17_ were dominant in Chilean mussels^55^ and salmon,^56^ whereas PCAs-C_>17_ were less abundant, a pattern resembling the urban PCA profiles in our study and suggesting comparable usage across the continent.

### Implication of the Study

This is not the first time that elevated concentrations of anthropogenic pollutants have been observed at high altitudes. However, the present study addresses a data gap by incorporating a nearby urban source as a reference point. Very counterintuitively, distant sources may exert an even greater influence on PCA levels and patterns at the summit than the nearby urban source. This high-altitude area may be more strongly influenced by distant sources than by the nearby city of 1.8 million residents, located just 16 km away, and this could also be the case of other mountains not only from Bolivia but from neighboring countries.

The enrichment of PCAs-C_14–17_ and PCAs-C_>17_ at high altitudes suggests that pollutants accumulating in mountain environments are less constrained by their physicochemical properties than might be expected based on the mountain cold-trapping model.^57^ Given the evidence that microplastics^53^ are also capable of long-range atmospheric transport, chemicals that are resistant to photodegradation, or those bound to particulates, including additives leaching from microplastics, could also be transported and become enriched in high-altitude regions.

Ultimately, high-altitude regions act as sinks for anthropogenic pollutants, where OC-normalized concentrations can be comparable to those in urban environments, and the Andean region is no exception to this. Although the dispersion range of PCAs-C_10–13_ and PCAs-C_14–17_ from urban sources at high altitudes does not exceed that in flat terrain, the altitudinal gradient allows long-range transported pollutants to influence a significantly broader range of high-altitude environments. Notably, PCAs represent only one fraction of the diverse chemical mixtures accumulating in these environments, raising concerns about environmental injustice for high-altitude communities and ecosystems. Identifying pollutant sources is crucial, but so is understanding how large-scale emissions and efficient transport mechanisms deposit such vast quantities of contaminants in these elevated yet vulnerable mountain regions.

## Supplementary Material



## Notes

Sampling sites (Figure S1), urban dispersion patterns based on dry-weight concentrations (Figure S2), linear fitting curves for dry-weight concentrations (Figure S3), linear regression analysis (Figure S4), raw data (Table S1), standard list for PCA (Table S2), modeled dry weight PCA concentrations (Table S3), C_10–16_ linear correlations (Table S4), modeled concentration gradients (Table S5), and fitting equations (Equations S1–S16) (PDF)
