# Supplementary material for: Polychlorinated Alkane Profiles and Concentrations in Bolivian Andes Soils Point to a Long-Range Transport Influence
Source: Environ Sci Technol. 2026 Mar 11;60(11):8618–27. doi: 10.1021/acs.est.5c14672 (PMC13019679; doi:10.1021/acs.est.5c14672)
Supplement: Supplementary file 1 [file es5c14672_si_001.pdf]

Supporting Information

Polychlorinated Alkane Profiles and Concentrations in Bolivian Andes Soils Point to a Long-Range Transport Influence

Bo Yuan[1,2*], Cheng Wu[3], Cynthia A. de Wit[1], Claudia Mohr[4,5], Marcos Andrade[6,7], Isabel Moreno[6,8], Volker Brüchert[9,10], Rienk Smittenberg[11], Matthew MacLeod[1]

[1] Department of Environmental Science (ACES), Stockholm University, Stockholm 106 91, Sweden

[2] Department of Chemistry and Biomedical Science, Norwegian University of Science and Technology (NTNU), Trondheim 7491, Norway

*bo.yuan@ntnu.no

[3] Department of Chemistry and Molecular Biology, Atmospheric Science, University of Gothenburg, Gothenburg 413 90, Sweden

[4] Department of Environmental Systems Science, ETH Zürich, Zürich 8092, Switzerland

[5] PSI Center for Energy and Environmental Research, Paul Scherrer Institute, Villigen 5232, Switzerland

[6] Laboratory for Atmospheric Physics, Institute for Physics Research, Universidad Mayor de San Andrés, La Paz, Bolivia

[7] Department of Atmospheric and Oceanic Sciences, University of Maryland, College Park, Maryland, 20742, USA

[8] Instituto de Investigaciones Fármaco Bioquímicas, Universidad Mayor de San Andrés, Av. Saavedra N 2224, La Paz, Bolivia

[9] Department of Geological Sciences, Stockholm University, Stockholm 106 91, Sweden

[10] Bolin Centre for Climate Research, Stockholm University, Stockholm 106 91 Sweden

[11] Swiss Federal Institute for Forest, Snow and Landscape Research, Birmensdorf 8903, Switzerland

This Supporting Information contains:

- **Total number of pages:** 11
- **Text sections:** 1
- **Figures:** 4 (Figures S1-S4)
- **Tables:** 5 (Tables S1-S5)
- **Equations:** 16

Text S1. Urban Transport Patterns based on Dry-weight Concentrations. The highest PCA concentrations were detected in the urban soils, with means (± standard

deviation) of 0.73 ± 0.53 ng/g dry weight (d.w.) for PCAs-C_{6-9}, 16.0 ± 10.8 ng/g d.w. for PCAs-C_{10-13}, 36.8 ± 26.0 ng/g d.w. for PCAs-C_{14-17}, and 7.68 ± 5.84 ng/g d.w. for PCAs-$C_{>17}$, respectively. Dry-weight concentrations were specifically used for urban transport analysis because previous PCA studies in urban soils have consistently reported their data on a dry-weight basis. Following the approach of Xu et al., who applied dry weight-based modeling to describe the dispersion of PCAs-C_{10-13} and PCAs-C_{14-17} from a PCA production plant into surrounding surface soils,[1] the dry weight soil concentrations were fitted to a Gaussian distribution model (r^2 = 0.28–0.71, $p < 0.05$, Figure S2). Unlike dispersion patterns observed by Xu et al. in flat, near-sea-level terrain where PCA concentrations remained low and stable within a 2–4 km distance of the emission source,[1] our study showed that the concentrations of PCAs-C_{6-9} and PCAs-C_{10-13} remained appreciable at similar distances and declined clearly, by 16% and 12% per km, respectively, beyond the urban boundary. In contrast, the less volatile PCAs (PCAs-C_{14-17} and PCAs-$C_{>17}$) remained stable, with concentration changes of less than 0.2% per km beyond the urban boundary (Table S1).

From 12 km onwards, observed PCA-C_{6-9} concentrations (0.273 ± 0.184 ng/g d.w., Table S3) closely followed the prediction from the Gaussian distribution model (0.265 ng/g d.w.), whereas concentrations of the other PCA groups deviated beyond the model's 95% confidence intervals. Notably, at this distance, where altitudes reached 4,800 meters and above, measured concentrations of PCAs-C_{10-13}, PCAs-C_{14-17}, and PCAs-$C_{>17}$ were significantly higher than the modeled values (one-sample t-test, $p < 0.05$). The dry weight concentrations suggested only weak tendencies with altitude, which were not statistically significant ($r^2 < 0.1$, $p > 0.1$, Figure S3).

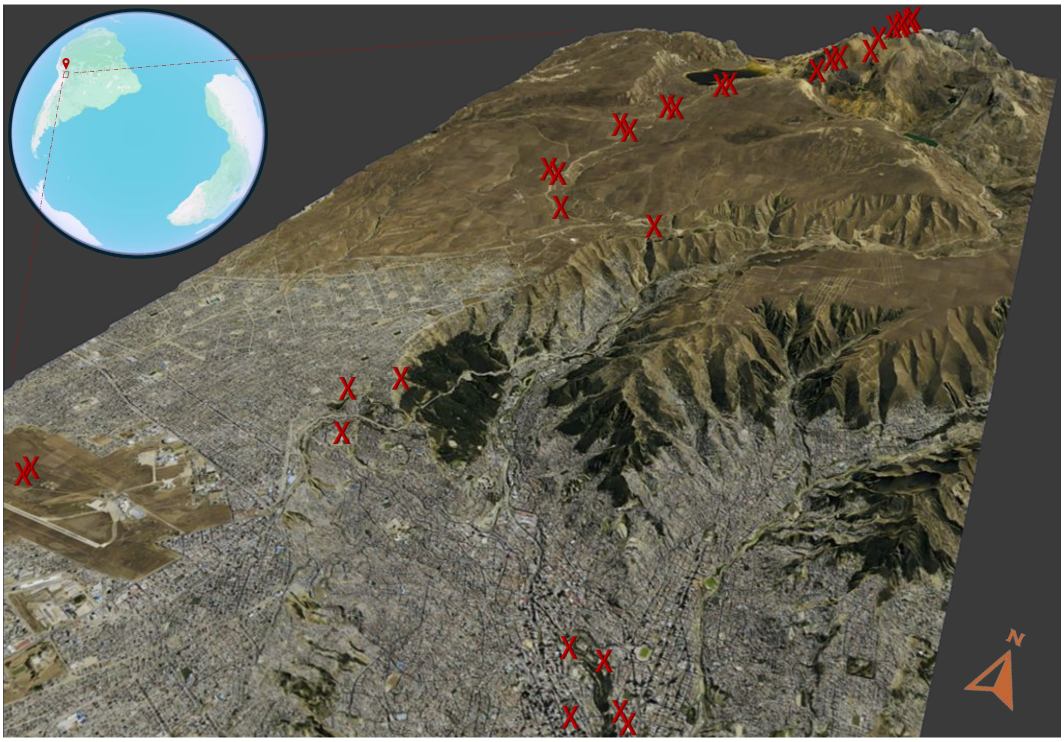

Figure S1. Map of soil sampling sites. Locations are marked with X. The figure provides a three-dimensional visualization of the study area for spatial context, but does not include quantitative elevation or terrain data.

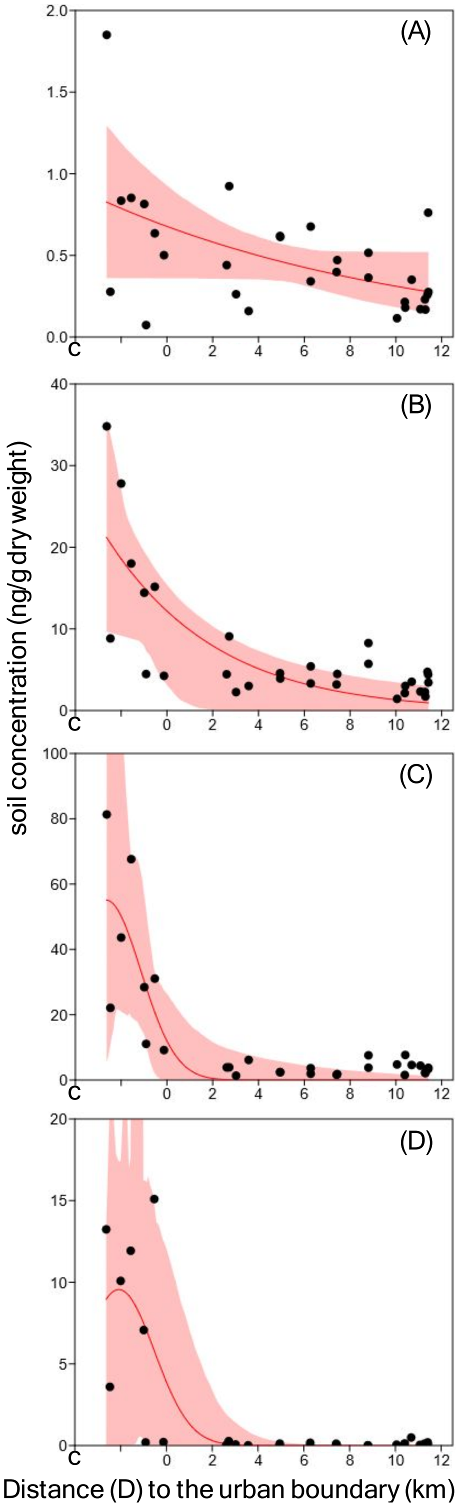

Figure S2. Soil concentrations (ng/g dw) of (A) PCAs-$C_{6\text{-}9}$, (B) PCAs-$C_{10\text{-}13}$, (C) PCAs-$C_{14\text{-}17}$, and (D) PCAs-$C_{>17}$ as a function of the distance from the urban boundary. Black dots indicate individual soil samples, red curves show Gaussian fits to the data, and shaded pink areas represent the 95% confidence intervals. The letter "c" on the horizontal axis denotes the center of the studied urban area. The distance between "c" and the urban boundary (0) was 4 km. For the fitted Gaussian equations, see Equations S1–S4 in the SI.

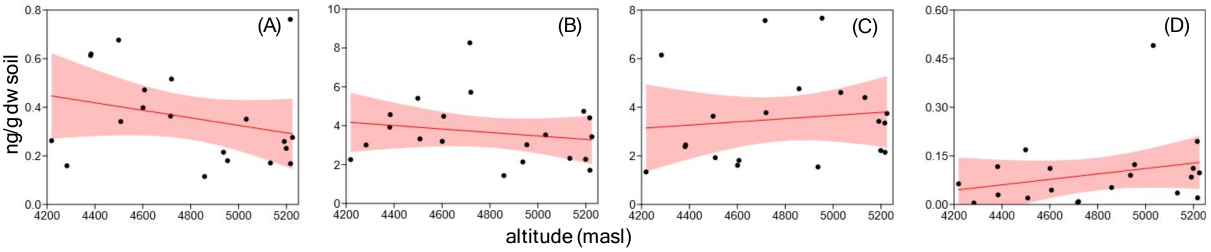

Figure S3. Soil concentrations (ng/g dw) of (A) PCAs-C_{6-9}, (B) PCAs-C_{10-13}, (C) PCAs-C_{14-17}, and (D) PCAs-$C_{>17}$ as a function of altitude, with linear fitting curves and the corresponding 95% confidence range. For fitted regression equations, see Equations S13–S16, respectively.

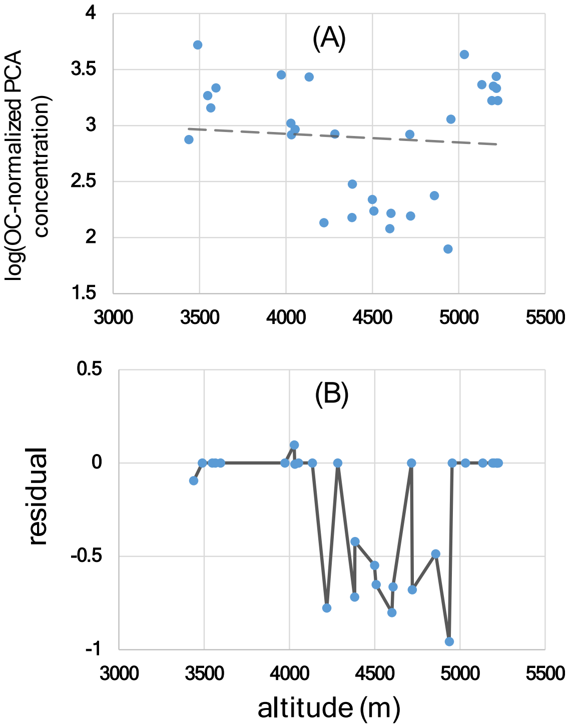

Figure S4. (A) Linear regression analysis between log-transformed OC-normalized sumPCA concentrations and altitude. The dashed line represents the linear fitting curve ($r^2 < 0.01$), indicating a weak linear relationship. (B) Residual plot showing a systematic, non-random deviation from zero across the altitude gradient. Residuals decrease from values close to zero to predominantly negative values and subsequently return toward zero, indicating a non-linear pattern not captured by a simple linear model. The center of this negative deviation is located at approximately 4700 masl.

Table S1. Soil sampling site information and PCA concentrations. The first section presents details of the sampling locations, including total organic carbon (TOC), total organic nitrogen (TON), and the distances from each site to the center of the eight urban sampling sites.

Category	Water content (%)	Altitude (m.a.s.l.)	GPS coordinates		Distance (km)	$\delta^{13}C$ (‰)	TOC (%)	$\delta^{15}N$ (‰)	TON (%)
			Latitude (S)	Longitude (W)					
Urban	0.9%	3438	16.515387	68.116588	3.09	-22.78	2.11	9.33	0.23
	5.9%	3489	16.510516	68.120774	2.44	-22.11	1.88	8.02	0.20
	3.4%	3547	16.502754	68.126698	1.53	-25.57	1.88	8.81	0.19
	20.5%	3565	16.503720	68.128449	1.37	-18.36	9.15	11.99	0.90
	2.2%	3595	16.512981	68.127213	2.00	-21.14	3.81	9.08	0.32
	1.7%	3973	16.488415	68.164967	3.01	-18.64	1.79	8.20	0.21
	5.3%	4053	16.484891	68.168106	3.47	-26.29	6.70	11.37	0.57
	4.5%	4134	16.475722	68.167004	3.87	-25.34	0.52	5.74	0.11
Transect	5.9%	4028	16.511110	68.199145	6.72	-25.05	1.35	4.07	0.17
	5.9%	4031	16.509958	68.198369	6.61	-23.98	1.06	5.40	0.14
	5.1%	4219	16.431983	68.150519	7.02	-25.92	2.88	5.96	0.28
	6.1%	4283	16.432042	68.167450	7.57	-24.25	1.11	7.30	0.11
	23.8%	4382	16.420914	68.174865	8.95	-25.56	4.66	3.01	0.40
	7.4%	4384	16.420904	68.174584	8.94	-24.39	2.56	8.41	0.26
	16.1%	4499	16.404623	68.169583	10.28	-24.84	4.52	6.71	0.45
	10.9%	4508	16.404570	68.169380	10.28	-25.61	3.26	4.14	0.30
	9.7%	4601	16.392616	68.167984	11.41	-24.90	4.43	5.79	0.42
	10.3%	4607	16.392397	68.168240	11.44	-24.56	4.14	6.58	0.40
	7.7%	4716	16.376208	68.157205	12.80	-25.15	1.95	7.92	0.19
	32.9%	4720	16.376230	68.156874	12.80	-25.17	6.43	6.78	0.58
	11.6%	4858	16.362656	68.141306	14.05	-23.93	2.69	7.60	0.25
	20.3%	4937	16.359356	68.139012	14.39	-24.71	5.04	4.65	0.46
	7.8%	4954	16.359209	68.138588	14.41	-24.24	0.96	6.17	0.12
	6.8%	5032	16.356576	68.134678	14.69	-24.63	0.21	4.58	0.12
	6.4%	5133	16.352765	68.136540	15.07	-23.99	0.30	6.25	0.13
Summit	4.7%	5191	16.349964	68.132494	15.38	-23.73	0.51	5.37	0.10
	5.3%	5199	16.351035	68.132118	15.27	-24.60	0.22	5.76	0.17
	5.2%	5216	16.349706	68.131552	15.41	-24.18	0.32	5.84	0.14
	7.0%	5217	16.350880	68.131491	15.29	-25.12	0.19	6.13	0.18
	5.9%	5225	16.349685	68.130666	15.42	-25.78	0.45	4.45	0.12

Table S1 (continued). Soil sampling site information and PCA concentrations. The second section provides PCA concentrations for individual soil samples, expressed on both a dry weight and organic carbon (OC)-normalized basis.

Category	Altitude (m.a.s.l.)	Distance* (km)	Dry weight PCA concentration (ng/g d.w.)				OC-normalized PCA concentration (ng/g OC)			
			C_{6-9}	C_{10-13}	C_{14-17}	$C_{>17}$	C_{6-9}	C_{10-13}	C_{14-17}	$C_{>17}$
Urban	3438	-0.91	0.0732	4.47	11.0	0.191	3.48	212	524	9.05
	3489	-1.56	0.852	18.0	67.6	11.9	45.3	957	3600	634
	3547	-2.47	0.277	8.83	22.1	3.59	14.8	470	1180	191
	3565	-2.63	1.85	34.8	81.3	13.2	20.2	381	889	145
	3595	-2.00	0.835	27.8	43.6	10.1	21.9	730	1150	265
	3973	-0.99	0.815	14.4	28.4	7.07	45.4	805	1590	395
	4053	-0.53	0.635	15.2	31.1	15.1	9.48	226	464	225
	4134	-0.13	0.501	4.25	9.17	0.212	96.2	815	1760	40.7
Transect	4028	2.72	0.924	9.08	3.89	0.270	68.4	672	288	20.0
	4031	2.61	0.440	4.44	3.86	0.0344	41.5	418	364	3.24
	4219	3.02	0.262	2.25	1.34	0.0637	9.09	78.1	46.4	2.21
	4283	3.57	0.159	3.01	6.15	<0.015	14.3	271	554	<1.11
	4382	4.95	0.613	3.92	2.38	0.117	13.2	84.2	51.1	2.51
	4384	4.94	0.619	4.57	2.45	<0.016	24.2	178	95.7	<2.56
	4499	6.28	0.676	5.41	3.63	0.169	15.0	120	80.3	3.73
	4508	6.28	0.341	3.32	1.92	0.0198	10.5	102	59.1	0.61
	4601	7.41	0.398	3.19	1.61	0.111	8.99	72.1	36.4	2.51
	4607	7.44	0.471	4.48	1.81	0.0444	11.4	108	43.7	1.07
	4716	8.80	0.364	8.26	7.57	<0.015	18.7	424	389	<1.95
	4720	8.80	0.516	5.72	3.78	<0.018	8.02	88.9	58.7	<6.43
	4858	10.05	0.115	1.43	4.76	0.0522	4.28	53.3	177	1.94
	4937	10.39	0.215	2.13	1.54	0.0900	4.26	42.3	30.7	1.79
	4954	10.41	0.180	3.02	7.66	0.123	18.7	313	795	12.8
	5032	10.69	0.351	3.52	4.61	0.491	169	1690	2210	236
	5133	11.07	0.171	2.32	4.40	0.0354	57.0	776	1470	11.8
Summit	5191	11.38	0.259	4.74	3.42	0.0843	50.8	929	670	16.5
	5199	11.27	0.231	2.27	2.22	0.111	107	1050	1030	51.7
	5216	11.41	0.762	4.41	3.35	0.194	240	1390	1060	61.2
	5217	11.29	0.168	1.71	2.15	0.0204	89.3	908	1140	10.8
	5225	11.42	0.276	3.43	3.74	0.0975	61.1	760	829	21.6

*Distance to the urban boundary.

Table S2. PCA reference standard list.

Quantifying	*Product Name*	*Manufacturer*	*Country*	*Ingredients and chlorine content**
**PCAs-$C_{6\text{-}9}$, very-short-chain**	CP-52	Unknown	China	PCAs-$C_{6\text{-}29}$† 52% Cl
**PCAs-$C_{10\text{-}13}$, short-chain**	Witaclor 149	Dynamit Nobel AG	Germany	PCAs-$C_{10\text{-}13}$ 49% Cl
	SCCP 51.5 %Cl	Ehrenstorfer GmbH	Germany	PCAs-$C_{10\text{-}13}$ 51.5% Cl
	SCCP 55.5 %Cl	Ehrenstorfer GmbH	Germany	PCAs-$C_{10\text{-}13}$ 55.5% Cl
	SCCP 63.0 %Cl	Ehrenstorfer GmbH	Germany	PCAs-$C_{10\text{-}13}$ 63.0% Cl
	Hüls 70C	Hüls AG	Germany	PCAs-$C_{10\text{-}13}$ 70% Cl
**PCAs-$C_{14\text{-}17}$, medium-chain**	MCCP 42.0 %Cl	Ehrenstorfer GmbH	Germany	PCAs-$C_{14\text{-}17}$ 42.0% Cl
	MCCP 52.0 %Cl	Ehrenstorfer GmbH	Germany	PCAs-$C_{14\text{-}17}$ 52.0% Cl
	MCCP 57.0 %Cl	Ehrenstorfer GmbH	Germany	PCAs-$C_{14\text{-}17}$ 57.0% Cl
	Cloparin 49st	Caffaro	Italy	PCAs-$C_{14\text{-}17}$ 49% Cl
	Cloparin 50	Caffaro	Italy	PCAs-$C_{14\text{-}17}$ 50% Cl
	Cereclor S52	INEOS Chlor Ltd.	UK	PCAs-$C_{14\text{-}17}$ 52% Cl
**PCAs-$C_{>17}$, long-chain**	Hüls 40N	Hüls AG	Germany	PCAs-$C_{18\text{-}26}$ 40% Cl
	Witaclor 549	Dynamit Nobel AG	Germany	PCAs-$C_{18\text{-}25}$ 49% Cl
	Uniclor40	Neville Chemical Co	USA	PCAs-$C_{22\text{-}27}$ 40% Cl
	LCCP 36.0 %Cl	Ehrenstorfer GmbH	Germany	PCAs-$C_{18\text{-}20}$ 36.0% Cl
	LCCP 49.0 %Cl	Ehrenstorfer GmbH	Germany	PCAs-$C_{18\text{-}20}$ 49.0% Cl

* Specifications from the manufacturer. The actual chain length distributions may extend beyond the specified range.

† Determined using an APCI-QTOF; not specified by the manufacturer.

Table S3. Modeled dry weight soil concentrations and concentration changes of PCAs, along with observed concentrations in relation to distance from the urban boundary.

PCA	Modeled concentration at the studied urban center (ng/g d.w.)	Modeled concentration at distance to the urban boundary (ng/g d.w.)						Concentration changes per km*			
		2 km	4 km	6 km	8 km	10 km	12 km	2-4 km	4-6 km	6-8 km	8-10 km
C6-9	0.914	0.582	0.500	0.427	0.365	0.311	0.265	-18.2%	-15.7%	-13.6%	-11.7%
C10-13	29.0	7.50	4.69	2.91	1.79	1.09	0.660	-19.3%	-12.3%	-7.7%	-4.8%
C14-17	37.4	0.510	0.0039	0.000	0.000	0.000	0.000	-2.7%	-0.02%	0.00%	0.00%
C>17	4.44	0.302	0.0044	0.000	0.000	0.000	0.000	-13.4%	-0.20%	0.00%	0.00%
	Mean ± SD concentration at the urban center (ng/g d.w.) (*n* = 8)	Mean ± SD concentration at sites located >12 km from the urban boundary (ng/g d.w.) (*n* = 10)									
C6-9	0.73 ± 0.53						0.273 ± 0.184				
C10-13	16.0 ± 10.8						2.898 ± 1.12				
C14-17	36.8 ± 26.0						3.79 ±1.75				
C>17	7.68 ± 5.84						0.130 ± 0.136				

*Calculated as the difference in modeled concentrations between a farther point and a location 2 km closer to the urban center, normalized to the modeled concentration at the urban center, and divided by 2 km.

Table S4. Linear correlations between OC-normalized concentrations of individual PCA carbon chain lengths and altitude, for samples collected at or above 4,600 masl.

Carbon	10	11	12	13	14	15	16
Slope	0.585	0.528	0.307	0.249	1.160	0.452	0.158
R^2	0.633	0.653	0.519	0.173	0.513	0.377	0.212
p	<0.01	<0.01	<0.01	0.12	<0.01	<0.05	0.10

Table S5. Modeled altitudinal concentration gradients of PCAs and combined concentration gradients relative to distance from the urban boundary. Concentration gradients were calculated based on the difference in modeled concentrations between farther and closer distances, normalized to the latter.

PCA	Modelled concentration gradient based on Gaussian fitting for urban dispersion			Modeled concentration gradient based on exponential fitting as a function of altitude*			Overall concentration gradient		
	2-4 km	4-6 km	6-8 km	4200-4400 masl	4400-4600 masl	4600-4800 masl	2-4 km	4-6 km	6-8 km
C6-9	86%	86%	85%	124%	146%	174%	107%	125%	149%
C10-13	63%	62%	61%	169%	174%	178%	106%	108%	109%

* $C_{PCA-C6-9} = 2.2569 \times 10^{-8} \times e^{0.0042738 \cdot A}$ +6.3815 (A=4200-5250 masl r^2=0.47, p<0.05)
$C_{PCA-C10-13} = 1.6813 \times 10^{-4} \times e^{0.003007 \cdot A}$ +9.5428 (A=4200-5250 masl r^2=0.61, p<0.05)

Fitting Equations

Eq.	Equation	in Figure
S1	$C_{PCA-C6-9} = 1076 \times e^{-(D+194.41)^2/5127.8}$ (r^2=0.28, p<0.05)	**S2A**
S2	$C_{PCA-C10-13} = 1337200 \times e^{-(D+109.11)^2/1025.88}$ (r^2=0.58, p<0.05)	**S2B**
S3	$C_{PCA-C14-17} = 54.732 \times e^{-(D+2.586)^2/4.299}$ (r^2=0.71, p<0.05)	**S2C**
S4	$C_{PCA-C>17} = 9.551 \times e^{-(D+0.0796)^2/4.8182}$ (r^2=0.63, p<0.05)	**S2D**
S5	$\log(C_{PCA-C6-9}) = -0.00063156 \cdot A + 3.95$ (r^2=0.39, p<0.05)	**3A**
S6	$\log(C_{PCA-C6-9}) = 0.0018631 \cdot A - 7.795$ (r^2=0.57, p<0.01)	**3A**
S7	$\log(C_{PCA-C10-13}) = -0.0010168 \cdot A + 6.6075$ (r^2=0.65, p<0.01)	**3B**
S8	$\log(C_{PCA-C10-13}) = 0.0018242 \cdot A - 6.5403$ (r^2=0.57, p<0.01)	**3B**
S9	$\log(C_{PCA-C14-17}) = -0.0015587 \cdot A + 8.7924$ (r^2=0.71, p<0.01)	**3C**
S10	$\log(C_{PCA-C14-17}) = 0.002196 \cdot A - 8.3791$ (r^2=0.62, p<0.01)	**3C**
S11	$\log(C_{PCA-C>17}) = -0.0019363 \cdot A + 8.8641$ (r^2=0.69, p<0.01)	**3D**
S12	$\log(C_{PCA-C>17}) = 0.0021698 \cdot A - 9.8306$ (r^2=0.54, p<0.01)	**3D**
S13	$C_{PCA-C6-9} = -0.00015494 \cdot A + 1.1001$ (r^2=0.08, p=0.23)	**S3A**
S14	$C_{PCA-C10-13} = -0.00089265 \cdot A + 7.9352$ (r^2=0.04, p=0.43)	**S3B**
S15	$C_{PCA-C14-17} = 0.00065733 \cdot A + 0.37333$ (r^2=0.01, p=0.61)	**S3C**
S16	$C_{PCA-C>17} = 0.000083993 \cdot A - 0.30909$ (r^2=0.07, p=0.27)	**S3D**

D: Distance to the urban boundary (km)
A: Altitude (masl)

Reference

(1) Xu, J.; Gao, Y.; Zhang, H.; Zhan, F.; Chen, J. Dispersion of Short- and Medium-Chain Chlorinated Paraffins (CPs) from a CP Production Plant to the Surrounding Surface Soils and Coniferous Leaves. *Environ. Sci. Technol.* **2016**, *50* (23), 12759–12766.
DOI: 10.1021/acs.est.6b03595.
